# Longitudinal Changes in Ultrasound-Assessed Femoral Cartilage Thickness in Individuals from 4 to 6 Months Following Anterior Cruciate Ligament Reconstruction

**DOI:** 10.1177/19476035211038749

**Published:** 2021-08-12

**Authors:** Caroline Lisee, Matthew Harkey, Zachary Walker, Karin Pfeiffer, Tracey Covassin, Jeffrey Kovan, Katharine D. Currie, Christopher Kuenze

**Affiliations:** 1 Department of Exercise and Sport Science, University of North Carolina at Chapel Hill, NC, USA; 2 Department of Kinesiology, Michigan State University, East Lansing, MI, USA; 3 Department of Orthopedics, Michigan State University, East Lansing, MI, USA; 4 College of Osteopathic Medicine, Michigan State University, East Lansing, MI, USA

**Keywords:** knee injury, minimal detectable change, semi-automated, segmentation

## Abstract

**Objective:**

Diagnostic ultrasound provides a valid assessment of cartilage health that has been used to observe cross-sectional cartilage thickness differences post-ACLR (anterior cruciate ligament reconstruction), but has not been used longitudinally during early recovery post-ACLR.

**Design:**

The purpose of this study was to assess longitudinal changes in femoral cartilage thickness via ultrasound in individuals at 4 to 6 months post-ACLR and compared to healthy controls. Twenty participants (50% female, age = 21.1 ± 5.7 years) completed testing sessions 4 and 6 months post-ACLR. Thirty healthy controls (57% female, age = 20.8 ± 3.8 years) without knee injury history completed 2 testing sessions (>72 hours apart). Femoral cartilage ultrasound images were captured bilaterally in ACLR participants and in the dominant limb of healthy controls during all sessions. Average cartilage thicknesses in the medial, intercondylar, and lateral femoral regions were determined using a semi-automated processing technique.

**Results:**

When comparing cartilage thickness mean differences or changes over time, individuals post-ACLR did not demonstrate between limb differences (*P*-range = 0.50-0.92), limb differences compared to healthy controls (*P*-range = 0.19-0.94), or changes over time (*P*-range = 0.22-0.72) for any femoral cartilage thickness region. However, participants demonstrated cartilage thickening (45%) or thinning (35%) that exceeded minimal detectable change (MDC) from 4 to 6 months post-ACLR, respectively.

**Conclusions:**

Using MDC scores may help better identify within-subject femoral cartilage thickness changes longitudinally post-ACLR due to bidirectional cartilage thickness changes.

## Introduction

Approximately one third of individuals with anterior cruciate ligament (ACL) injury and ACL reconstruction (ACLR) demonstrate tibiofemoral or patellofemoral osteoarthritis (OA) within 10 years of injury.^1,2^ It is imperative to identify individuals who demonstrate early changes in knee joint health post-ACLR to determine which individuals may be at increased risk for OA and may benefit from OA prevention strategies before irreversible tissue damage has occurred. Radiographic and magnetic resonance imaging (MRI) are used to help categorize the severity of OA in clinical setting.^3-8^ However, radiographic imaging is limited because it is unable to directly assess articular cartilage that is impacted during early phases of OA progression.^9^ While MRI is able to quantitatively assess articular cartilage thickness, serial assessments of MRI over short periods of time are cost-prohibitive. As a result of these barriers, there is a lack of follow-up imaging that occurs during the first year after ACLR despite the fact that one third of patients will display MRI evidence of OA by 1 year post-ACLR.^2^

Diagnostic ultrasound is an emerging tool used to assess knee structural pathology and is a clinically accessible alternative to radiographic or MRI. Ultrasound assessments allow for safe and cost-effective serial assessments during the first 6 months after ACLR when patients are likely to be engaged in consistent encounters with health care professionals and when the knee may be most responsive to OA prevention interventions.^10,11^ Ultrasound is a valid^12^ and reliable^13^ tool for quantifying femoral cartilage thickness. A previous cross-sectional study in individuals ranging from 7 to 103 months post-ACLR reported greater medial femoral cartilage thickness assessed via ultrasound in the ACLR limb compared to their contralateral limb and the dominant limb of uninjured controls.^14^ However, serial ultrasound assessment of femoral cartilage thickness has not been conducted during the first 6 months after ACLR while patients remain engaged in the health care system. While it is recommended that patients delay return to unrestricted activity at least 9 months after ACLR,^15,16^ previous research suggests that patients are discharged from rehabilitation on average 6 months post-ACLR^17^ and may be cleared for unrestricted activity as early as 4 months post-ACLR.^18^ Therefore, it is necessary to determine if femoral cartilage thickness changes between 4 and 6 months post-ACLR. Assessment of cartilage thickness changes may help identify individuals with early knee joint health changes during this early period of recovery when patients are likely to remain in consistent contact with a healthcare provider.

There is conflicting evidence regarding the direction of femoral cartilage thickness change (i.e., thinning or thickening) after ACLR and in the progression of OA. It is well accepted that late-stage OA is characterized by cartilage thinning.^19^ However, articular cartilage thickness may also increase during early OA progression after injury as a result of cartilage swelling, especially within the medial femoral cartilage.^20-22^ After ACLR, individuals demonstrate medial femoral cartilage thickening, and femoral trochlea cartilage thinning on MRI from 3 to 12 months post-ACLR.^23,24^ Therefore, cartilage thickness changes may differ depending on the timing of assessment and which cartilage region is assessed. Studies assessing femoral cartilage thickness via ultrasound after ACLR are also conflicting, suggesting that both greater and lesser involved limb femoral cartilage thickness may be present 3 and 5 years post-ACLR when compared to the contralateral limb.^14,25^ It is necessary to characterize which patterns of cartilage thickening or thinning occur over time in individuals after ACLR and whether cartilage thickness differs compared to uninjured populations.

The purpose of this study was 2-fold: (1) to compare femoral cartilage thickness assessed via ultrasound between the involved limb and contralateral limb of individuals at 4 and 6 months post-ACLR and (2) to compare femoral cartilage thickness in the involved limb and contralateral limb of individuals recovering from ACLR to the dominant limb of healthy controls. We hypothesized that individuals would demonstrate increased medial femoral cartilage thickness in the involved limb from 4 to 6 months post-ACLR as well as greater medial femoral cartilage thickness in the involved limb compared to their contralateral limb. We also hypothesized that individuals with ACLR will demonstrate greater involved limb medial femoral cartilage thickness compare to healthy controls, but there will be no differences when comparing the contralateral limb to a healthy control limb.

## Methods

Femoral cartilage thickness was assessed via ultrasound in individuals with a history of ACLR and healthy controls over 2 study visits in this longitudinal cohort study. Individuals with a history of ACLR attended visits at 4 months (± 2 weeks) and 6 months (± 2 weeks) post-ACLR, and healthy controls without a history of ACLR attended 2 visits at least 72 hours apart. This study was approved by Michigan State University’s Institutional Review Board, and all participants ≥18 years old provided written informed consent before engaging in study activities. All participants under the age of 18 provided informed assent and their parents or guardians provided informed consent prior to engaging in any study-related procedures.

### Participants

Participants with a history of ACLR were recruited from a local sports medicine clinic where they were treated by 1 of 4 fellowship-trained orthopedic surgeons, and from the university community via flyers, emails, and word of mouth. A convenience sample of healthy participants were also recruited through flyers, emails, and word of mouth on the university campus and were not matched to ACLR participants based on demographic criteria. Participants with ACLR and healthy controls were included if they were between the ages of 16 and 35 years old. Inclusion criteria for participants with ACLR also included primary, unilateral ACLR within the past 4 months, and self-reported regaining full knee flexion range of motion. We did not exclude participants with ACLR if they had other surgical procedures (meniscal or MCL injury or related surgical procedures) completed at the time of ACLR. Study personnel completed reviews of available surgical charts (*N* = 16, 80%) to confirm concomitant diagnoses and surgical procedures in participants with ACLR and are reported in **Table 1**. Participants with ACLR and healthy controls were excluded if they had a previous history of intraarticular knee injury or surgery not related to the current ACLR, lower extremity orthopedic injury in the past 6 weeks, rheumatoid arthritis, or any other chronic illnesses that may impede their ability to complete the tasks required of the study.

**Table 1.** Participant Characteristic Comparisons between Individuals with a History of ACLR at 4 Months Postsurgery and Healthy Controls.

Participant Characteristics	ACLR Group at 4 Months (*n* = 20)	Healthy Controls (*n* = 30)	*P*
Age (years)	21.1 ± 5.7	20.8 ± 3.8	0.60
BMI (kg/m^2^)	26.4 ± 6.1	24.7 ± 4.0	0.23
Sex (% female)	50% (*n* = 10)	57% (*n* = 17)	0.26
Activity level	8.8 ± 1.4	7.2 ± 1.6	<0.001
Graft type	10 HT/9 BPTB/1 ALLO	—	—
Meniscal injury^a^	63% (*n* = 10)	—	—
Meniscectomy^a^	25% (*n* = 4)	—	—
Meniscal repair^a^	50% (*n* = 8)	—	—
MCL injury^a^	13% (*n* = 2)	—	—
Chondral injury^a^	13% (*n* = 2)	—	—
Chondroplasty^a^	13% (*n* = 2)	—	—

ACLR = anterior cruciate ligament reconstruction; BMI = body mass index; HT = hamstring tendon; BPTB = bone-patellar tendon-bone; ALLO = allograft.

a Data only available for 16 participants with ACLR.

### Resting Cartilage Ultrasound Imaging Assessment: Visit 1

All participants were required to provide a urine sample to assess their hydration status. A participant’s hydration status was defined using urine specific gravity (USG) assessed via an Atago 3730 digital refractometer (ATAGO U.S.A., Inc., Bellevue, WA) because previous research suggests that dehydration may negatively impact articular cartilage imaging.^26^ Participants who were dehydrated (USG > 1.025)^27^ were rescheduled to eliminate hydration status as a potential confounding factor.

Participants were seated with knees in an extended position for 30 minutes to minimize the effects of knee joint loading experienced during activities of daily living prior to the assessment.^28,29^ Three ultrasound images of anterior femoral cartilage were captured bilaterally using a transverse suprapatellar approach by a single assessor with a Vivid iQ ultrasound machine and 12L-RS linear probe (GE Healthcare, Boston, MA) using a valid^12,30^ and reliable^14,28,29,31^ assessment technique. In brief, participants were instructed to sit with their backs flat against the wall and bend their knee to 140° of knee flexion as determined by a manual goniometer. The distance between the posterior aspect of the calcaneus of the flexed knee and the wall were recorded using a tape measure affixed to the table. A participant’s limb was placed in the same position for follow-up assessments based on the distance between the calcaneus and the wall to ensure similar knee flexion placement that allows for consistently imaging the same location on the femoral cartilage. To image the femoral cartilage, the ultrasound probe was placed perpendicular to the anterior surface of the femoral condyles and aligned with the most anterior aspects of the medial and lateral femoral condyles, superior to the patella (Suppl. Fig. 1).^29^ A transparency grid placed over the monitor display of the image was used to record the position of the medial and lateral femoral condyles. We confirmed a similar position of these structures on the transparency grid to improve reliability of knee images between sessions.^29^ For participants with a history of ACLR, ultrasound images were collected in the contralateral limb followed by the involved limb. The contralateral limb was consistently imaged first to avoid participants’ apprehension in placing the involved knee in large angles of knee flexion by demonstrating the position in the non-surgical limb.

At the end of the session, all participants completed the Tegner Activity Scale to measure pre-injury level of activity on a scale of 0 to 10 in individuals post-ACLR and current level of activity in healthy control.^32^ A score of 10 indicates high level of activity based on participation in a competitive sport at national or elite levels and a score of 0 indicates inability to work or participate in activity due to knee problems.

### Resting Cartilage Ultrasound Imaging Assessment: Visit 2

Healthy control participants returned for a second visit after at least 72 hours to determine test-retest reliability between visits. While not a primary aim of this study, test-retest reliability has not been previously established for this technique. Therefore, it was essential to establish acceptable reliability prior to assessing change over time in a clinical population. Participants with a history of ACLR returned for a second visit at 6 months post-ACLR to track outcomes over time. The hydration screening process and resting ultrasound imaging assessment described at the visit 1 were repeated in visit 2.

### Ultrasound Image Processing

**Figure 1.**
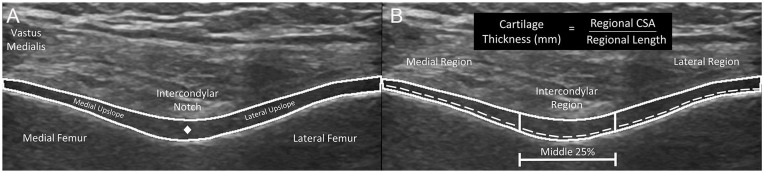
(**A**) Total cross-sectional area (CSA) of femoral cartilage is outlined by the white line and the center point of the cartilage is represented by the diamond. (**B**) Solid outlined portions of the cartilage represent cross-sectional area of medial, intercondylar, and lateral regions and the dotted line represents the length of the cartilage bone-interface for each region. Medial, intercondylar, and lateral cartilage thickness (mm) is calculated by dividing the cartilage CSA of each region by the length of each region.

Ultrasound images were processed using open-source ImageJ software (National Institute of Health, Bethesda, MD). Femoral cartilage images were deidentified and randomized by a study team member (ZW) and processed by a different rater (CL) who was blinded to participant grouping, time point, and limb. Total femoral cartilage cross-sectional area (CSA) was measured as the space between the superior synovial-cartilage border and the inferior cartilage-bone border (**Fig. 1A**).^28,29,31^ The central point of the femoral cartilage was manually identified by the rater as the middle of the synovial-cartilage border of the cartilage separating the medial and lateral upslopes (**Fig. 1A**). After segmenting the total CSA and the central point of the femoral cartilage, the segmented images were processed through a custom MATLAB code (Version 9.2, Mathworks, Natick, MA) to separate the total femoral cartilage region into standardized medial, intercondylar, and lateral regions. The intercondylar region was defined as the middle 25% of the femoral cartilage extending from the manually identified central point (**Fig. 1B**). The medial region was defined from the medial border of the intercondylar region to the medial border of the image. The lateral region length was defined from the lateral border of the intercondylar region to the lateral border of the image. The MATLAB program also automatically calculated the cartilage length in each region as the length of the cartilage-bone interface within each region. Average femoral cartilage thickness (the primary outcomes of interest for each image) was calculated in the medial, intercondylar, and lateral regions by dividing the regional CSA (mm^2^) by the cartilage length (mm) in each region (**Fig. 1B**).

### Sample Size Estimation

The sample size for this study (ACLR *n* = 20, Healthy *n* = 30) was similar to a cross-sectional study comparing femoral cartilage thickness assessed via ultrasound between the involved limb and contralateral limb in individuals with a history of ACLR (months since surgery = 37.0 ± 26.6) as well as the limb of healthy controls (ACLR *n* = 20, Healthy *n* = 28).^14^ Therefore, based on these previous studies, this justifies that our sample size should be large enough to detect medium to large effects (Cohen’s *d* = 0.46-0.79)^14^ for cartilage thickness differences between limbs in our study.

### Statistical Analysis

#### Comparison of Participant Characteristics between Groups

Descriptive statistics (i.e., means and standard deviations) were calculated for all demographic outcomes and medial, intercondylar, and lateral femoral cartilage thickness outcomes. Independent *t*-tests or Fisher exact tests were used to compare participant characteristics between individuals with a history of ACLR at 4 months postsurgery and healthy controls and determine if the groups had similar demographic characteristics at enrollment. All analyses were performed using SPSS Statistics (version 26, IBM Corp, Armonk, NY).

#### Test-Retest Reliability and Precision of Average Cartilage Thickness in Healthy Participants

We have previously established intra- and interrater reliability of assessing cartilage thickness on images acquired using the semi-automated technique.^13^ However, to further highlight the utility of this technique separate intraclass correlation coefficients (ICC_2,k_) were calculated to determine test-retest reliability of each regional femoral cartilage thickness in the dominant limb of healthy controls between the first and second visits by a single assessor (CL). ICC values are classified as poor (ICC < 0.49), moderate (ICC = 0.5-0.74), good (ICC = 0.75-0.89) and excellent (ICC > 0.9).^33^

#### Comparisons of Femoral Cartilage Thickness between Time, Limbs, and Groups

For the primary analysis, 2-way repeated-measures analysis of variance (ANOVA) were used to compare cartilage regional thickness between limbs (involved limb and contralateral limb) and over time (4 and 6 months post-ACLR) in individuals with a history of ACLR. Significant interactions were further investigated using a paired sample *t*-test to identify differences between limbs at each time point and within limbs across time. Independent *t*-tests were also used to compare cartilage thickness between the involved and contralateral limbs of individuals with ACLR with the dominant limb of healthy controls from the first study visit. Alpha was set to 0.05 *a priori*.

#### Frequency of Femoral Cartilage Thickness Exceeding the Minimal Detectable Change in ACLR Participants

Since both cartilage thickening or thinning may occur in these individuals, group averages may confound the results and prevent observations of patient-specific changes in cartilage thickness. Standard error of measurement (SEM) and minimal detectable change based on 90% confidence (MDC_90_)^29,34^ were also calculated to determine the precision of change in femoral cartilage outcomes for test-retest reliability.

$$SEM=\mathrm{Standard}\;\mathrm{Deviation}\sqrt{1-ICC}$$

$$MDC_{90}=1.654\times SEM\times \sqrt{2}$$

As an exploratory analysis, we determined the frequency of knees that exceeded the MDC_90_ for an increase or decrease in each cartilage thickness region from 4 to 6 months post-ACLR to identify if individual patients are experiencing meaningful cartilage change.

## Results

### Comparison of Participant Characteristics between Groups

All participants recovering from ACLR (*n* = 20) completed both visits at 4 and 6 months post-ACLR (range of days between visits = 42 to 86), and all healthy control participants (*n* = 30) completed the follow-up visit to assess reliability at least 3 days after the initial visit (range of days between visits = 3 to 13). The ACLR group demonstrated greater pre-injury activity levels compared to the current activity level of healthy controls (*P* < 0.001). There were no significant differences in age, sex, or BMI between participants with ACLR and healthy controls indicating that these groups were similar at enrollment (Table 1).

### Test-Retest Reliability and Precision of Average Cartilage Thickness in Healthy Participants

ICCs, SEM, and MDC_90_ outcomes are reported in **Table 2**. Test-retest reliability between visits 1 and 2 for average femoral cartilage thickness was excellent for all regions (ICC_2,k_ = 0.97-0.99).

**Table 2.** Test-Retest (ICC_2,k_ and 95% Confidence Intervals), Standard Error of Measurement (SEM), and Minimal Detectable Change (MDC) for Femoral Cartilage Thickness.

Average Cartilage Thickness	Test-Retest Reliability		
	ICC	SEM	MDC
Medial (mm)	0.97 [0.97, 0.95]	0.07	0.16
Intercondylar (mm)	0.99 [0.98, 0.99]	0.06	0.14
Lateral (mm)	0.98 [0.97, 0.99]	0.05	0.12

### Comparisons of Femoral Cartilage Thickness between Time, Limbs, and Groups

There were no significant limb main effects (*P* = 0.50-0.92), time main effects (*P* = 0.22-0.72), or limb by time interactions (*P* = 0.24-49) for femoral cartilage thickness in any region between the involved and contralateral limbs at 4 or 6 months post-ACLR (Suppl. Fig. 2). There were no significant differences between either limb in individuals with ACLR at 4 or 6 months post-ACLR when compared to the dominant limb of healthy controls (**Table 3**).

**Table 3.** Femoral Cartilage Thickness (mm) at 4 and 6 Months Post-ACLR (Mean ± Standard Deviation).

Cartilage Region	Limb	4 Months Post-ACLR	6 Months Post-ACLR	4 Month Comparison with Healthy Control	6 Month Comparison with Healthy Control
Medial thickness (mm)	Involved	2.04 ± 0.59	2.13 ± 0.56	*P* = 0.36	*P* = 0.81
	Contralateral	2.01 ± 0.39	2.05 ± 0.37	*P* = 0.19	*P* = 0.29
	Healthy control	2.16 ± 0.38		—	—
Intercondylar thickness (mm)	Involved	2.53 ± 0.52	2.54 ± 0.48	*P* = 0.84	*P* = 0.84
	Contralateral	2.61 ± 0.78	2.52 ± 0.44	*P* = 0.88	*P* = 0.77
	Healthy control	2.57 ± 0.61		—	—
Lateral thickness (mm)	Involved	2.05 ± 0.29	2.08 ± 0.32	*P* = 0.97	*P* = 0.47
	Contralateral	2.12 ± 0.35	2.07 ± 0.32	*P* = 0.72	*P* = 0.78
	Healthy control	2.04 ± 0.36		—	—

### Frequency of Femoral Cartilage Thickness Exceeding the MDC in ACLR Participants

Based on the change exceeding the MDC_90_, the frequency of knees demonstrating an increase, decrease, or no change in cartilage thickness from 4 to 6 months post-ACLR are reported in **Table 4**. Forty-five percent and 35% of participants experienced cartilage thickening or thinning that exceeded MDC_90_ from 4 to 6 months post-ACLR in the involved limb, respectively. In the contralateral limb, 20% and 25% of participants experienced cartilage thickening or thinning that exceeded MDC_90_ from 4 to 6 months post-ACLR, respectively.

**Table 4.** Individuals with Changes in Average Cartilage Thickness That Exceeded the MDC_90_ in the Involved and Contralateral Limbs from 4 to 6 Months Post-ACLR.

Participant	Involved Limb			Contralateral Limb		
	Medial	Intercondylar	Lateral	Medial	Intercondylar	Lateral
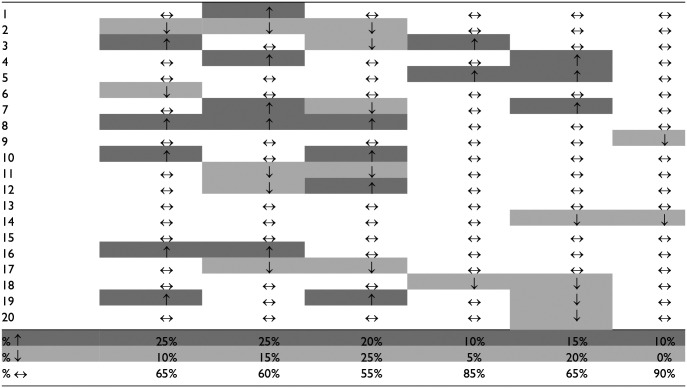						

↑ = cartilage thickening (dark grey), ↓ = cartilage thinning (light grey), and ↔ = no change (white) in average cartilage thickness that exceeded MDC_90_ from 4 to 6 months post-ACLR.

## Discussion

The results of our analysis indicate that femoral cartilage thickness did not demonstrate mean differences between limbs in individuals 4 to 6 months post-ACLR or compared to the limbs of healthy controls. Additionally, femoral cartilage thickness did not change bilaterally from 4 to 6 months post-ACLR. However, the lack of cartilage thickness change may be due to participants with ACLR experiencing both cartilage thickening and thinning over the 2-month observation period. Therefore, individual participants experience cartilage thickness change that exceeds measurement error, but the bi-directionality (i.e., both thickening and thinning) in the direction of change between participants may have masked these changes in our primary analyses that focused on group means.

While the results of our primary analysis suggest a lack of mean thickness changes over time, thickness changes that exceed MDC occurred in both limbs of many participants after ACLR, especially within the involved limb. Cartilage thickness changes were heterogeneous in directionality and regional location in participants from 4 to 6 months post-ACLR. A total of 45% of individuals demonstrated cartilage thickening, and 35% demonstrated cartilage thinning in at least one region of the involved limb, while only 20% and 25% of individuals demonstrated cartilage thickening and thinning in the contralateral limb, respectively (**Table 4**). These results are similar to previously reported bidirectionality of regional cartilage thickness changes on MRI from 3 to 12 months post-ACLR^24^ and in individuals prior to the onset of accelerated knee OA.^35^ Therefore, conducting analyses based on group means may be an inadequate assessment of thickness changes longitudinally due to the bidirectionality of cartilage changes. Cartilage thickness analyses that conduct thickness change assessment based on MDC may help overcome limitations of bidirectional cartilage thickness changes that cancel out each other when there is thickening and thinning in the same regions across participants and may be more helpful in quantifying changes in articular cartilage structure over time. While this study is the first to our knowledge to assess longitudinal ultrasound-based femoral cartilage thickness changes in individuals post-ACLR, lack of long-term follow-up is a limitation of the study and implications about long-term knee joint health cannot be concluded. Approximately one third of patients post-ACLR demonstrate early osteoarthritis as measured by MRI within the year post-ACLR.^2^ Future studies should complete longitudinal studies that extend to at least 12 post-ACLR to determine if ultrasound-based femoral cartilage thickness changes that exceed MDC are associated with worsening knee joint health related to OA development and provide prognostic benefits.

Meaningful changes in cartilage thickness indicating either cartilage thickening and thinning of the involved limb were evident 4 to 6 months post-ACLR in almost half of the participants. It remains unclear why thickening occurs in some individuals after ACLR while thinning occurs in others based on ultrasound and MRI assessment during the first year post-ACLR. In OA disease progression, cartilage thinning results from degeneration of extracellular matrix components including aggrecan and type II collagen. In comparison, increases in cartilage thickness are hypothesized to result from cartilage swelling from an influx of water.^20,36^ Compositional MRI studies have highlighted involved limb cartilage changes in proteoglycan density and type II collagen orientation during the first 6 months post-ACLR.^37-39^ Femoral cartilage thickness changes in the contralateral limb that exceeded MDC in some participants were unexpected, but we speculate that cartilage changes may be associated with bilateral aberrant gait biomechanics present within the first 6 months post-ACLR that affect cartilage loading. At 6 months post-ACLR, bilateral gait biomechanics differ between individuals with ACLR compared to uninjured controls matched based on age, sex, and BMI^40^ and have also been associated with worse femoral cartilage composition.^38,41^ Cartilage thickness is a measure of cartilage macrostructure and it is unclear how ultrasound-based cartilage thickness and compositional changes are related. While not included as an outcome in the current study, ultrasound echo-intensity which quantifies image pixel intensity may serve as a complementary outcome to cartilage thickness in ultrasound assessment. Ultrasound echo-intensity is associated with extracellular water content of muscles in aging populations^42^ and may provide insight into compositional changes of cartilage water content. Future research should explore the relationships between ultrasound assessment of cartilage thickness and echo-intensity with MRI markers of cartilage composition after ACLR.

A limitation of this study is that the presence of concomitant meniscal surgical procedures or articular cartilage pathologies at time of ACLR surgery were not controlled for despite the fact that they may impact cartilage health.^43,44^ Approximately 63% of participants that had available surgical data also received a meniscectomy or meniscal repair surgery and 12.5% of participants had a chondroplasty at the time of ACLR. A larger sample size may be necessary to determine the effects of meniscal surgical procedures or articular cartilage pathologies impact resting femoral cartilage thickness. The sample size of the current study was similar to a previous cross-sectional study that reported significant differences between limb in individuals post-ACLR as well as compared to the limb of healthy controls.^14^ However, the lack of *a priori* power analysis limits our ability to determine if the sample size was large enough to detect between-limb differences. Another study limitation includes that the single assessor performing the ultrasound imaging was not blinded to group or time point which may add bias to the imaging assessment. We acknowledge that there are feasibility challenges to implementing assessor blinding to participant group and time point including that the surgical knee is easily identifiable by surgical scarring. Regardless, the single assessor performing the segmentation was blinded to reduce image processing bias.

When comparing overall group means, individuals recovering from ACLR did not demonstrate significant differences in femoral cartilage thickness assessed with ultrasonography between limbs, compared to healthy controls, or from 4 to 6 months after surgery. However, one third to one quarter of individuals demonstrated meaningful decreases or increases in involved limb femoral cartilage thickness 4 to 6 months post-ACLR. Ultrasound is an accessible assessment tool that identifies meaningful thickening and thinning in femoral cartilage thickness from 4 to 6 months post-ACLR.

## Supplemental Material

sj-pdf-1-car-10.1177_19476035211038749 – Supplemental material for Longitudinal Changes in Ultrasound-Assessed Femoral Cartilage Thickness in Individuals from 4 to 6 Months Following Anterior Cruciate Ligament Reconstruction Click here for additional data file. Supplemental material, sj-pdf-1-car-10.1177_19476035211038749 for Longitudinal Changes in Ultrasound-Assessed Femoral Cartilage Thickness in Individuals from 4 to 6 Months Following Anterior Cruciate Ligament Reconstruction by Caroline Lisee, Matthew Harkey, Zachary Walker, Karin Pfeiffer, Tracey Covassin, Jeffrey Kovan, Katharine D. Currie and Christopher Kuenze in CARTILAGE

sj-pdf-2-car-10.1177_19476035211038749 – Supplemental material for Longitudinal Changes in Ultrasound-Assessed Femoral Cartilage Thickness in Individuals from 4 to 6 Months Following Anterior Cruciate Ligament Reconstruction Click here for additional data file. Supplemental material, sj-pdf-2-car-10.1177_19476035211038749 for Longitudinal Changes in Ultrasound-Assessed Femoral Cartilage Thickness in Individuals from 4 to 6 Months Following Anterior Cruciate Ligament Reconstruction by Caroline Lisee, Matthew Harkey, Zachary Walker, Karin Pfeiffer, Tracey Covassin, Jeffrey Kovan, Katharine D. Currie and Christopher Kuenze in CARTILAGE

sj-xlsx-1-car-10.1177_19476035211038749 – for Longitudinal Changes in Ultrasound-Assessed Femoral Cartilage Thickness in Individuals from 4 to 6 Months Following Anterior Cruciate Ligament Reconstruction Click here for additional data file. sj-xlsx-1-car-10.1177_19476035211038749 for Longitudinal Changes in Ultrasound-Assessed Femoral Cartilage Thickness in Individuals from 4 to 6 Months Following Anterior Cruciate Ligament Reconstruction by Caroline Lisee, Matthew Harkey, Zachary Walker, Karin Pfeiffer, Tracey Covassin, Jeffrey Kovan, Katharine D. Currie and Christopher Kuenze in CARTILAGE This article is distributed under the terms of the Creative
Commons Attribution 4.0 License ( https://creativecommons.org/licenses/by/4.0/) which
permits any use, reproduction and distribution of the work without
further permission provided the original work is attributed as specified
on the SAGE and Open Access pages ( https://us.sagepub.com/en-us/nam/open-access-at-sage).
