# Supplementary figures and images for: Longitudinal Changes in Ultrasound-Assessed Femoral Cartilage Thickness in Individuals from 4 to 6 Months Following Anterior Cruciate Ligament Reconstruction
Source: Cartilage. 2021 Aug 12;13(1 Suppl):738S–746S. doi: 10.1177/19476035211038749 (PMC8808943; doi:10.1177/19476035211038749)

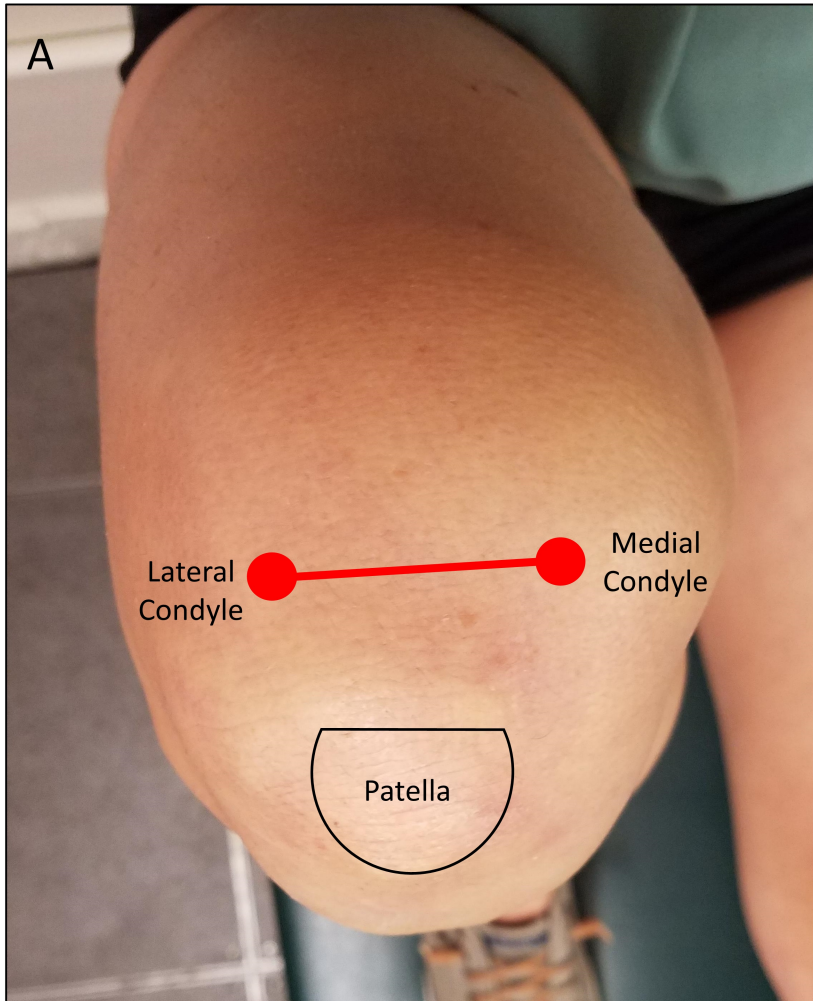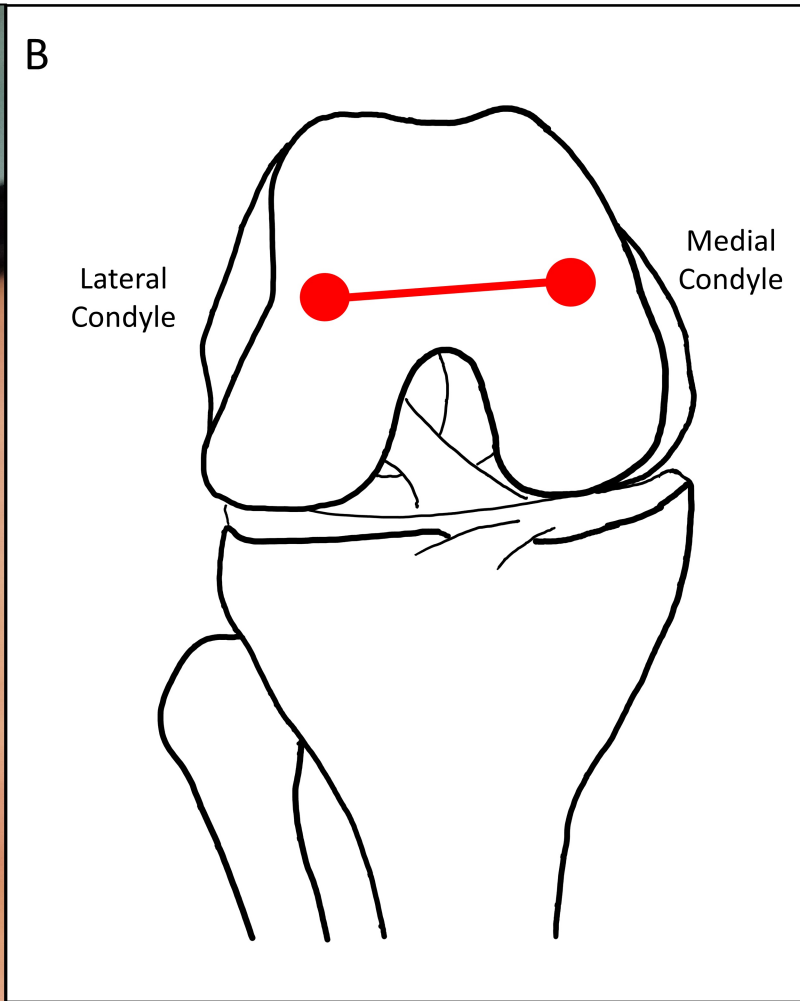

Supplement: sj-pdf-1-car-10.1177_19476035211038749 – Supplemental material for Longitudinal Changes in Ultrasound-Assessed Femoral Cartilage Thickness in Individuals from 4 to 6 Months Following Anterior Cruciate Ligament Reconstruction [file sj-pdf-1-car-10.1177_19476035211038749.pdf]

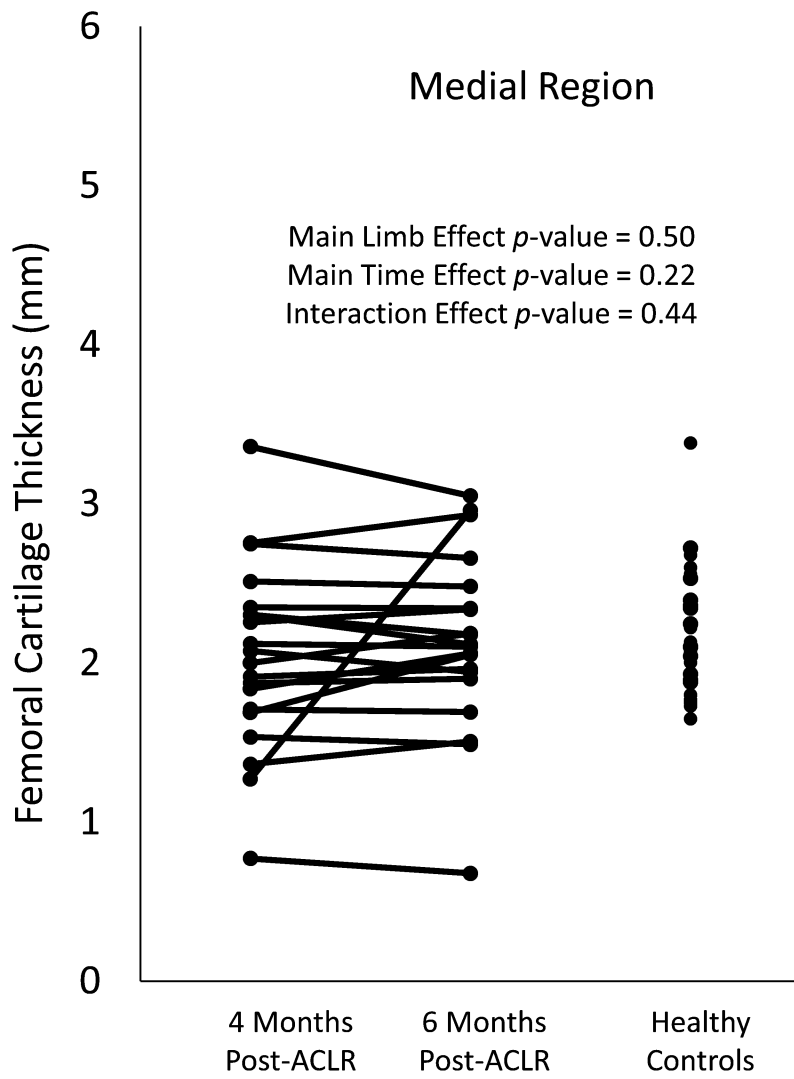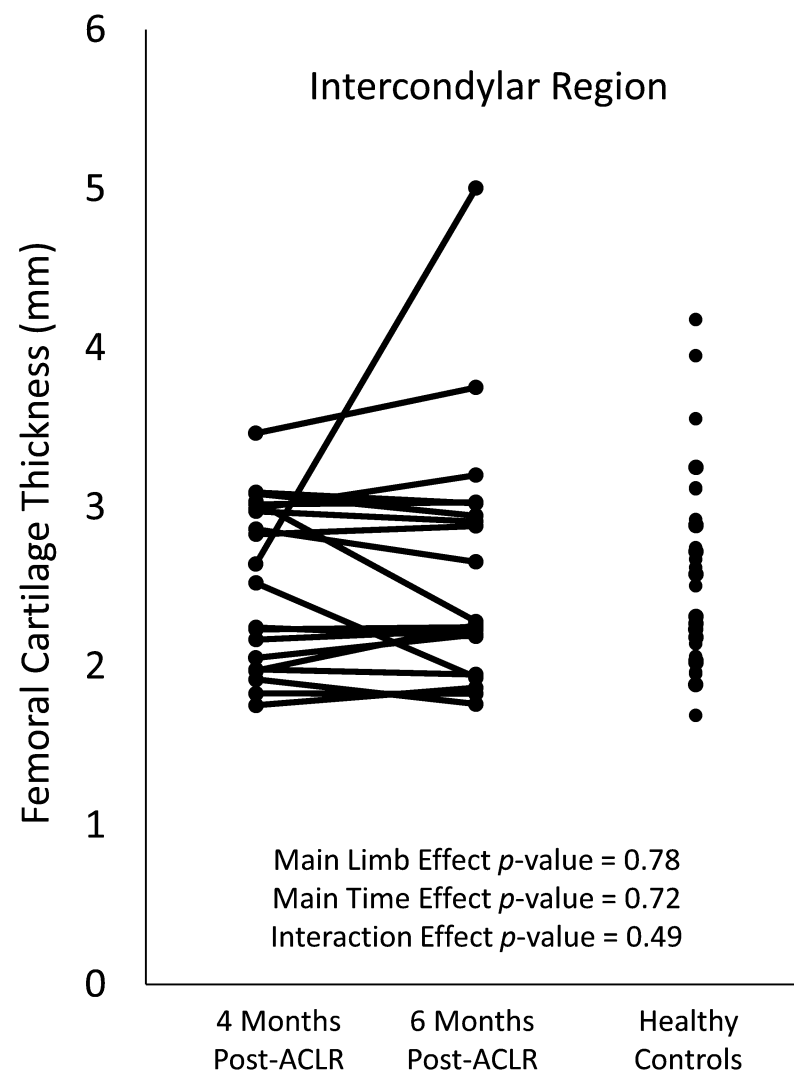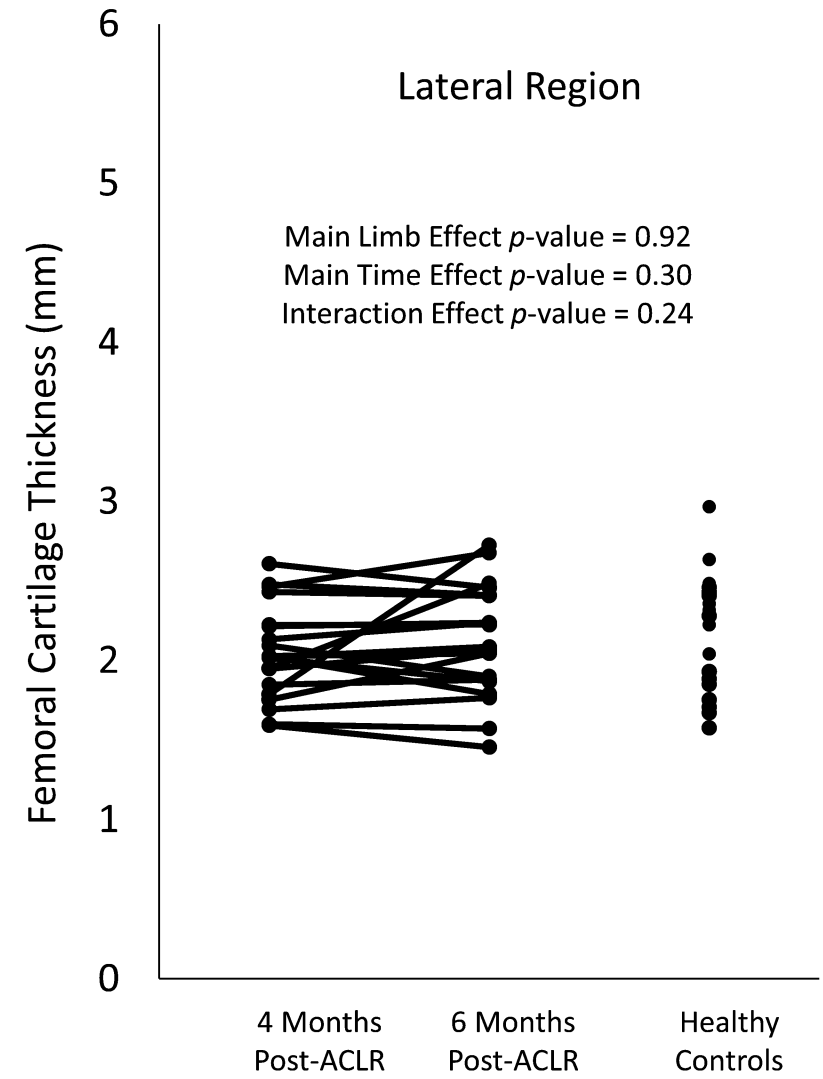

Supplement: sj-pdf-2-car-10.1177_19476035211038749 – Supplemental material for Longitudinal Changes in Ultrasound-Assessed Femoral Cartilage Thickness in Individuals from 4 to 6 Months Following Anterior Cruciate Ligament Reconstruction [file sj-pdf-2-car-10.1177_19476035211038749.pdf]
